# Effect of Population Heterogenization on the Reproducibility of Mouse Behavior: A Multi-Laboratory Study

**DOI:** 10.1371/journal.pone.0016461

**Published:** 2011-01-31

**Authors:** S. Helene Richter, Joseph P. Garner, Benjamin Zipser, Lars Lewejohann, Norbert Sachser, Chadi Touma, Britta Schindler, Sabine Chourbaji, Christiane Brandwein, Peter Gass, Niek van Stipdonk, Johanneke van der Harst, Berry Spruijt, Vootele Võikar, David P. Wolfer, Hanno Würbel

**Affiliations:** 1 Animal Models in Psychiatry, Central Institute of Mental Health (CIMH), Mannheim, Germany; 2 Animal Welfare and Ethology, University of Giessen, Giessen, Germany; 3 Behavioural Biology, University of Muenster, Muenster, Germany; 4 Animal Sciences, Purdue University, West Lafayette, Indiana, United States of America; 5 Psychoneuroendocrinology, Max Planck Institute of Psychiatry, Munich, Germany; 6 Delta Phenomics BV, Utrecht, The Netherlands; 7 Neuroscience Center, University of Helsinki, Helsinki, Finland; 8 Institute of Anatomy, University of Zürich, Zürich, Switzerland; Université Pierre et Marie Curie, France

## Abstract

In animal experiments, animals, husbandry and test procedures are traditionally standardized to maximize test sensitivity and minimize animal use, assuming that this will also guarantee reproducibility. However, by reducing within-experiment variation, standardization may limit inference to the specific experimental conditions. Indeed, we have recently shown in mice that standardization may generate spurious results in behavioral tests, accounting for poor reproducibility, and that this can be avoided by population heterogenization through systematic variation of experimental conditions. Here, we examined whether a simple form of heterogenization effectively improves reproducibility of test results in a multi-laboratory situation. Each of six laboratories independently ordered 64 female mice of two inbred strains (C57BL/6NCrl, DBA/2NCrl) and examined them for strain differences in five commonly used behavioral tests under two different experimental designs. In the standardized design, experimental conditions were standardized as much as possible in each laboratory, while they were systematically varied with respect to the animals' test age and cage enrichment in the heterogenized design. Although heterogenization tended to improve reproducibility by increasing within-experiment variation relative to between-experiment variation, the effect was too weak to account for the large variation between laboratories. However, our findings confirm the potential of systematic heterogenization for improving reproducibility of animal experiments and highlight the need for effective and practicable heterogenization strategies.

## Introduction

Experimental results that cannot be reproduced are scientifically worthless and a nuisance if published in the literature where they may create uncertainty and hinder scientific progress. Poor reproducibility and lack of external validity are an issue throughout laboratory research from mass spectrometry proteomic profiling [1] and microarray analysis [2]–[5] to the social and behavioral sciences [6], [7]. In animal experiments, however, where the lives of animals are highly valuable, poor reproducibility is also an ethical issue. Thus, animal care and use regulations require scientists not to unnecessarily duplicate previous experiments [8]–[10]. This explicitly assumes that animal results are reproducible by different laboratories, and that duplication therefore represents unnecessary animal use. However, a review of the scientific literature casts serious doubt on this assumption, indicating that poor reproducibility may be rather widespread [11]–[22].

In animal experiments, animals, housing and experimental conditions are traditionally standardized to render the animals' responses to experimental treatments more homogeneous, thereby reducing within-experiment variation and increasing test sensitivity [23], [24]. Because higher test sensitivity allows a reduction of sample size, standardization is also promoted for ethical reasons as a mean to reduce animal use [25], [26]. Moreover, standardization across experiments is assumed to reduce between-experiment variation, thereby improving reproducibility among laboratories [24], [27]. However, by reducing within-experiment variation, standardization may limit inference to the specific experimental conditions [28], [29]. Given that most biological traits exhibit environmental plasticity [30], different experimental conditions may produce different experimental outcomes. Because laboratories inherently vary in many experimental features (e.g. experimenter, room architecture), conditions are generally more homogenous within than between laboratories. Therefore, standardization inevitably induces disparity between results from different laboratories. In contrast, controlled variation of experimental conditions may render the animals within experiments more heterogeneous, thereby improving the external validity and hence the reproducibility of experimental results [28], [29], [31].

Indeed, we have recently shown in mice that standardization may increase the incidence of spurious results in behavioral tests, accounting for poor reproducibility between replicate experiments, while systematic variation of experimental conditions (heterogenization) attenuated spurious results, thereby improving reproducibility [32]. However, our findings were challenged because they were based on retrospective analysis, and because heterogenization may be logistically unfeasible [33]. We therefore tested standardization against a simple form of heterogenization for reproducibility across four independent replicate experiments. Systematic variation of only two factors was sufficient to mimic the range of differences between the replicate experiments, resulting in almost perfect reproducibility [34].

In a real multi-laboratory situation, however, between-experiment variation might be considerably greater. Recent multi-laboratory studies revealed large effects of the laboratory as well as strong interactions between genotype and the laboratory environment [13], [17], [35], [22]. To investigate whether simple forms of heterogenization within laboratories render populations of mice sufficiently heterogeneous to guarantee robust results across laboratories, we designed a multi-laboratory study involving six laboratories, and compared the effect of standardization against heterogenization on the reproducibility of behavioral differences between two common inbred strains of mice. Although heterogenization significantly increased within-experiment variation relative to between-experiment variation, the effect was too weak to account for the large variation between laboratories and improve reproducibility substantially. Thus, further research is needed to establish effective and practicable heterogenization strategies.

## Methods

### Experimental design

**Figure 1:**
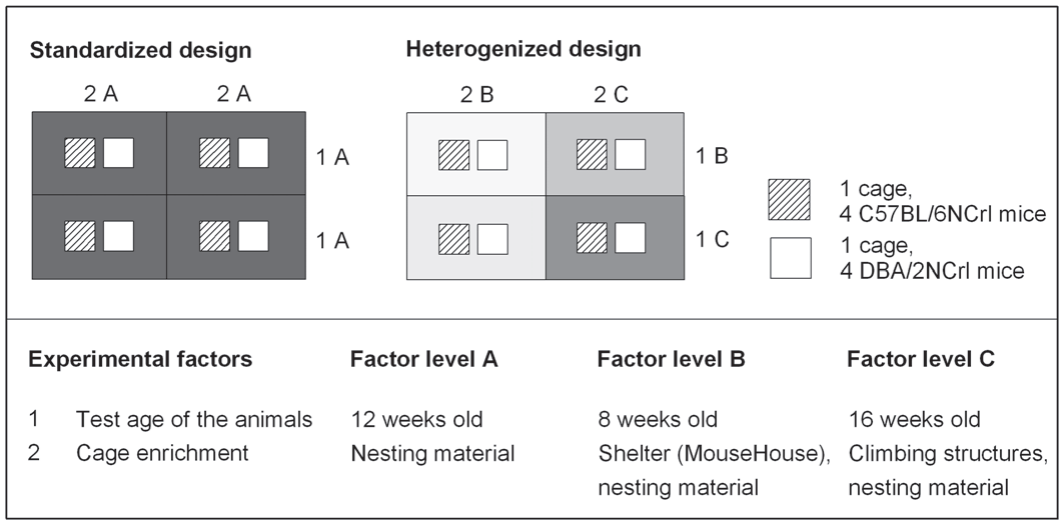
Experimental design. Each of six laboratories used 64 female mice of two inbred strains (C57BL6NCrl, DBA/2NCrl) ordered in two consecutive batches (n = 16 per batch and strain), with each batch being allocated to one experimental design. Upon arrival of a batch, the 16 mice per strain were randomly assigned to four cages in groups of four. To test heterogenization against standardization, we selected two experimental factors (test age, cage enrichment) and chose three factor levels A, B and C for each factor. Within each laboratory, the two factors were either standardized to factor level A (standardized design, uniform grey) or systematically varied across B and C using a 2×2 factorial design (heterogenized design, varying grey). According to the 2×2 factorial design of the heterogenized condition, study populations were divided into four blocks that were also characterized by similar microenvironmental differences due to cage position within the rack.

Each of six laboratories used 64 female mice of two inbred strains (C57BL6NCrl, DBA/2NCrl, n = 32 each) and examined them for strain differences in five commonly used behavioral tests (barrier test, vertical pole test, elevated zero maze, open field test, novel object test). To test heterogenization against standardization, each laboratory successively conducted the same experiment twice, using two different experimental designs with half of the mice allocated to each design. In the standardized design, experimental conditions were standardized, while they were systematically varied in the heterogenized design. For heterogenization, we selected two experimental factors (test age, cage enrichment) that typically vary between experiments in different laboratories, and chose three factor levels A, B and C for each factor (age: A = 12 weeks old, B = 8 weeks old, C = 16 weeks old; cage enrichment: A = nesting material, B = shelter, C = climbing structures). Within each laboratory, the two factors were standardized to factor level A in the standardized design and systematically varied across B and C using a 2×2 factorial design in the heterogenized design (Fig. 1). Because both age and enrichment have been demonstrated to affect and interact with a wide variety of potential outcome measures [36]–[42], heterogenization across these two factors was expected to create a range of different phenotypes within experiments, thereby increasing the external validity and thus the reproducibility of the results across the six laboratories.

Besides the two experimental factors that were standardized or heterogenized depending on the experimental design, the following factors were controlled and standardized in both experimental designs and all six laboratories: order of tests, test protocols, animal supplier, and housing protocols (number of animals/cage, housing period prior to testing, position of cages within the rack, interval of cage changes). All other variables varied between laboratories depending on laboratory standards. These included: details of the housing conditions (e.g. local tap water, food type, local bedding material, cage size), physical arrangement of housing and testing rooms (e.g. local room architecture, humidity, lighting, temperature), test apparatuses, tracking software (e.g. ANYmaze or EthoVision), experimenter, handling method (e.g. with/without gloves), identification method (e.g. ear punctures, fur markings) arrival and test dates, and test time (see Tables 1 and 2).

**Table 1:** Laboratory-specific housing conditions and animal care routines (STAN = standardized design, HET = heterogenized design).

	Giessen	Muenster	Zürich	Mannheim	Munich	Utrecht
**Arrival and test dates**						
**Arrival (STAN)**	Tue, 04/11/08	Tue, 09/06/09	Wed, 02/09/09	Wed, 06/05/09	Thu, 06/08/09	Wed, 03/06/09
**Arrival (HET)**	Tue, 11/11/08	Tue, 26/05/09	Wed, 26/08/09	Wed, 20/05/09	Thu, 13/08/09	Wed, 27/05/09
**Tests (STAN)**	Mon, 24/11/08	Mon, 29/06/09	Mon, 28/09/09	Mon, 01/06/09	Mon, 31/08/09	Mon, 29/06/09
**Tests (HET)**	Mon, 01/12/08	Mon, 15/06/09	Mon, 21/09/09	Mon, 15/06/09	Mon, 07/09/09	Mon, 22/06/09
**Housing conditions**						
**Food type**	Altromin 1324	Altromin 1324	Kliba Nafag 3430	Ssniff R/M-H	Altromin 1324	CRM (E) Expanded
**Bedding**	GRADE 6, Hellmann	Allspan, Höveler	Lignocel S3-4	Rehofix MK-2000	LTE E-001, ABEDD	Woodchips, ABEDD
**Cage size**	Type III	Type III	Type III	Type III	Type III	Type II elongated
**Physical arrangement of the housing room (HR)**						
**Humidity**	35±5%	60±5%	50±5%	50±5%	60±5%	67±10%
**Temperature**	21±1°C	20±1°C	21±1°C	20±0.2°C	21±1°C	21±1°C
**Lighting**	8–20 white light,	8–20 white light,	20–8 white light,	19–7 white light,	8–20 white light,	19–7 white light,
	20–8 lights off	20–8 lights off	8–20 lights off (rev.)	7–19 lights off	20–8 lights off	7–19 red light (rev.)
**Animal care**						
**Who?**	experimenter	experimenter	experimenters (2)	experimenters (2)	experimenters (2)	animal keeper
**Cage cleaning**	1/week, Friday	1/week, Tuesday	1/week, Wednesday	1/week, Wednesday	1/week, Friday	1/week, Monday
**Handling**	gloves, tail	gloves, tail	without gloves, tail	gloves, tail	gloves, tail	without gloves, tail
**Disturbance**	none	other mice in HR	other mice in HR	other mice in HR	other mice in HR	radio background
					(for first 10 days only)	
**Identification**	fur markings	fur markings	tails markings,	tail markings,	ear punctures	ear punctures, tail
			black marker	black marker		markings, black marker

**Table 2:** Laboratory-specific testing procedures and apparatuses.

	Giessen	Muenster	Zürich	Mannheim	Munich	Utrecht
**Behavioral testing**						
**Test room**	separate room	separate room	separate room	separate room	same as housing room	separate room
**Distance**	about 15m	about 30m	about 10m	about 1m	same room	about 10m
**Lighting**	all tests:	white light,	all tests:	red light,	white light	red light
	white light, 60 lx	OF: 120lx, EZM: 220lx	white light, 20lx	EZM: white light, 25lx	all tests: 60lx	
**Test time**	start: 9 a.m.,	start: 10 a.m.,	start 9–10 a.m.,	start: 10 a.m.,	start: 9 a.m.,	start: 9 a.m.,
	inactive phase	inactive phase	active phase	active phase	inactive phase	active phase
**Software**	EthoVision 3.1	ANYmaze	EthoVision 3.0	EthoVision XT	ANYmaze	EthoVision XT
**Cleaning**	30% Isopropanol	30% EtOH	water	70% EtOH	80% EtOH	water, cleaner
**Experimenter**	PhD student	PhD student	postdoc,	postdoc (EZM, OFT/NOT),	postdoc,	biotechnician
			biotechnician	biotechnician (BT, VPT),	student assistant	
				2 trainees (assistance)		
**Apparatuses**						
**BT**	Macrolon Type III,	Macrolon Type III,	Macrolon Type III	Macrolon Type III,	Macrolon Type III,	Macrolon Type III,
	barrier: 3cm high,	barrier: 3cm high,	barrier: 3cm high,	barrier: 1cm high,	barrier: 3cm high	barrier: 3cm high,
	0.6cm wide,	0.5cm wide,	0.5cm wide	0.6cm wide,	0.5cm wide	0.6cm wide,
	dark grey plastic	transparent plastic	dark grey plastic	transparent plastic	dark grey plastic	dark grey plastic
**VPT**	wooden pole, Ø2cm,	wooden pole, Ø2cm,	wooden pole, Ø2cm,	wooden pole, Ø2cm,	wooden pole, Ø2cm,	wooden pole, Ø2cm,
	length: 45cm	length: 45cm	length: 45cm	length: 45cm	length: 45cm	length: 45cm
**EZM**	light grey plastic,	grey plastic, covered	light grey plastic	grey plastic, covered	light grey plastic,	light grey plastic,
	elevated 40cm,	with a white plastic runway,	elevated 40cm,	with black cardboard paper	elevated 40cm,	elevated 40cm,
	Ø46cm, 5.5cm width	elevated 40cm,	Ø46cm, 5.5cm width	elevated 50cm,	Ø46cm, 5.5cm width	Ø46cm, 5.5cm width
		Ø46cm, 5.5cm width		Ø46cm, 6cm width		
**OFT+NOT**	4 adjacent dark grey	4 adjacent grey arenas	4 adjacent white	4 adjacent white	4 adjacent dark grey	rat phenotyper,
	plastic arenas	with white ground plates	plastic arenas	plastic arenas	plastic arenas	transparent plastic
	(50cm×50cm)	(40cm×40cm)	(50cm×50cm)	(50cm×50cm)	(50cm×50cm)	(45cm×45cm)

### Laboratories

The study was conducted in the following six laboratories: (1) Animal Welfare and Ethology, University of Giessen (H. Würbel), (2) Behavioural Biology, University of Muenster (N. Sachser), (3) Psychoneuroendocrinology, Max Planck Institute of Psychiatry, Munich (C. Touma), (4) Animal Models in Psychiatry, Central Institute of Mental Health, Mannheim (P. Gass), (5) Delta Phenomics B.V. in Utrecht (B. Spruijt) and (6) Institute of Anatomy, University of Zürich (D. Wolfer). Each lab provided space in a conventional colony room for animal housing and a test room for behavioral testing. Animal care was provided by each lab's animal care staff together with the designated experimenter of each laboratory who also implemented cage enrichments and conducted behavioral testing throughout the two test weeks. Experimenters were a PhD student in Giessen, a PhD student in Muenster, a postdoctoral research fellow and a student assistant in the Munich lab, a technician and a postdoctoral research fellow in Mannheim, a biotechnician in the Utrecht lab, and a postdoctoral research fellow and a biotechnician in Zürich. All experimenters were adept in working with mice and conducting behavioral tests. Experimenters were not blinded to strain, age, housing conditions and experimental design. However, because our outcome measure was reproducibility across laboratories and each laboratory had its own experimenter, the experimenters' knowledge about the animals and their expectations about the outcome of the study could not bias our outcome measure.

### Between-laboratory standardized conditions and procedures

#### Animals and housing conditions

**Figure 2:**
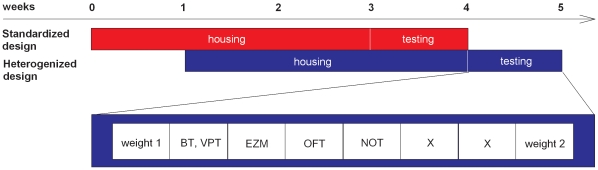
Experimental procedure followed by each laboratory. The 64 mice per laboratory, aged nine weeks for the standardized design, and five and thirteen weeks for the heterogenized design, were supplied in two independent batches (n = 16/strain). Upon arrival, the mice were group-housed in conventional polycarbonate cages for three weeks. Cages of the standardized design (red) contained two pieces of tissue paper (nesting material), while half of the cages of the heterogenized design (blue) contained a mouse house and the other half a climbing structure and a wooden ladder. Subsequent to the three-week housing phase, mice were subjected to a battery of five behavioral tests. The whole experimental procedure lasted five weeks, including a three-week housing phase, a one-week test phase and one week shift between the behavioral tests of the standardized and the heterogenized design. The order was balanced across the six laboratories with three laboratories starting with the standardized and three laboratories with the heterogenized design.

The 384 female mice (C57BL/6NCrl, DBA/2NCrl, n = 192 each) were obtained from Charles River Laboratories (Sulzfeld, Germany) aged nine weeks for the standardized condition, and aged five and thirteen weeks for the heterogenized condition (Fig. 2). Each lab independently ordered 32 females per strain that were supplied consecutively in two batches (n = 16/strain), one for the standardized design and one for the heterogenized design. The order of supply was balanced across laboratories with three laboratories (Giessen, Mannheim, Munich) starting with the standardized design and three laboratories (Muenster, Zürich, Utrecht) starting with the heterogenized design. Upon arrival, the mice were randomly assigned to same-strain groups of four and housed in conventional polycarbonate cages with sawdust, standard mouse diet and tap water *ad libitum*. Depending on the experimental design, cages contained additional equipment: Cages of the standardized design (A) additionally contained two soft tissue papers (Tork, SCA Hygiene Products GmbH, Wiesbaden, Germany), while half of the cages of the heterogenized design (B) contained a mouse house (MouseHouse, Tecniplast, Italy) and one tissue paper and the other half (C) a climbing structure (18 cm long, 10 cm high) [43], a wooden ladder (3 rungs, each 5 cm long, 14 cm high; Trixie Heimtierbedarf, Tarp, Germany) and one tissue paper. Cages were cleaned once per week, except for the test week to minimize disruption due to cage cleaning before testing. Mice were housed under these conditions for three weeks before the onset of the test phase (Fig. 2). Temperature and relative humidity were stable within laboratories, but differed between them (see Table 1). Similarly, all mice were held under a constant 12 h light-dark cycle, but the time schedules differed between laboratories (see Table 1).

Depending on the position in the rack, cages may differ in local environmental conditions (e.g. temperature, humidity, lighting, and disturbance) due to variation in proximity to ventilation, lights and human traffic. To avoid position bias, we controlled for cage position in the experimental design [44]. Thus, the eight cages of one design were stacked in two horizontal lines of four cages in one rack, with cages of DBA/2NCrl and C57BL/6NCrl mice balanced for horizontal and vertical position in the rack, and each vertical pair of cages of C57BL/6NCrl and DBA/2NCrl mice was treated as a block, assuming greater microenvironmental similarity within blocks than between blocks.

All procedures complied with the regulations covering animal experimentation within the EU (European Communities Council Directive 86/609/EEC) and in the countries in which the experiments were conducted (Germany: Deutsches Tierschutzgesetz; The Netherlands: Dutch Animal Welfare Act; Switzerland: Schweizerisches Tierschutzgesetz). They were conducted in accordance with the institutions' animal care and use guidelines and, where necessary, approved by the national and local authorities. The German labs (Giessen, Munich, Münster and Mannheim) did not need formal approval of the study by governmental authorities, because the study did not involve any harmful procedures. In the Utrecht lab, the study was approved by the Dutch Ethical Commission (Lely-DEC) under license number DPh-09-04, and the lab's permission to conduct animal experiments was granted by their general license number 24900 provided by the Dutch Government. In the Zürich lab, the study was approved by the Swiss Federal Veterinary Office under license number 204/2008. Moreover, all efforts were made to minimize the number of animals used and the severity of procedures applied in this study.

#### Behavioral testing

Mice were subjected to five behavioral tests that are commonly performed in drug-screening or behavioral phenotyping studies. They were conducted in the same order in all six laboratories: day 1: barrier test (BT), vertical pole test (VPT), day 2: elevated zero maze (EZM), day 3: open field test (OFT) and day 4: novel object test (NOT). To monitor health status, mice were weighed prior to and after testing (Fig. 2).

Testing order of cages was balanced across strain and rack position, and the mice of one cage were tested either simultaneously (OFT, NOT) or successively (BT, VPT, EZM). Apparatuses were cleaned with water or alcohol solution between trials.

#### Barrier test

To test the exploratory drive, mice were individually placed into an unfamiliar, empty type III Macrolon cage, divided in two halves by a plastic hurdle (see Table 2). At the beginning of each trial, a mouse was placed into one of the compartments according to a pseudo-random schedule. The test was finished when the mouse either crossed the barrier (all four paws on the other side of the barrier) or a maximum time of 300 s elapsed without the mouse climbing over the barrier. The latency to cross the barrier was used as measure of exploratory behavior.

#### Vertical pole test

The vertical pole test is a measure of motor coordination and balance that requires minimal equipment [45]. A wooden pole, approximately 2 cm in diameter and 40 cm long, was wrapped with cloth tape for improved traction. The mouse was placed on the centre of the pole that was held in a horizontal position by hand. The pole was then gradually lifted to a vertical position. The test was finished when the mouse either fell off the pole or held fast to it for 180 s. The latency to fall off the pole was used as dependent variable.

#### Elevated zero maze

On an EZM, the exploratory drive of mice is competing with their natural avoidance of heights and open spaces [45]. The EZM is a modification of the elevated plus maze that was first introduced and pharmacologically validated in rats [46], [47]. The advantage of the EZM is that it lacks the ambiguous central square of the traditional plus maze. The apparatus consisted of a circular platform, elevated 40–50 cm above the floor, divided into two open and two closed sectors enclosed by walls of about 20 cm height. At the beginning of each trial, the mouse was placed in one of the two closed sectors and behavior was recorded for 300 s. By using specialized tracking software, the total path moved on the maze as well as the path moved within, the time spent in, and the number of entries into the open and closed sectors, were automatically recorded. Moreover, head dips, stretched postures and rearing behavior were manually calculated according to the following definitions:

- *Head dip (HD)*: The animal dips its head over the side of the maze while its body remains on the maze.
- *Protected HD*: Head dips are considered protected when the animal dips its head over the side of the maze while its body remains in a closed segment.
- *Stretched posture*: Elongation of the body while maintaining the hind paws fixed, followed by retraction.
- *Rearing*: Standing upright on the hind limbs with or without touching a wall surface.

#### Open field test

The open field test is the most widely used behavioral test since it was developed by Hall [48], [49]. It has been validated pharmacologically as a test of anxiety [50], but is also used to measure exploratory and locomotor drive in laboratory rodents [45]. The apparatus consisted of an open box, 40 cm×40 cm minimum size, virtually divided into various zones (corners, 5 cm wall zone, centre). Mice were placed into the centre of the empty open field arena and videotracked for 10 min. The time spent in, the distance travelled within, and the number of entries into each zone, were calculated. In addition, the total distance moved during the 10 min session was analyzed and the number of fecal boli dropped was counted at the end of each trial.

#### Novel object test

In combination with an open field test, the novel object test serves to discriminate between approach and avoidance tendencies towards novel stimuli [51]. Twenty-four hours after the open field test, the animals were re-exposed for 10 min to the same arena with a novel object (black pine cone, autoclaved, about 7 cm high and 6 cm in diameter mounted on a metal plate; Miroflor, Greiz, Germany) placed upright in the centre of the arena. In addition to the zones defined for the open field test, two zones surrounding the object (15 cm, 25 cm diameter) were defined as exploration zones. The zone defined by the object itself was excluded from the exploration zones to avoid confounding “sitting on the object” with “object exploration”. Again, time spent in, distance travelled within, and the number of entries into the various zones were calculated. Object exploration time and frequency were assessed using the time spent in, and the frequency of entering the exploration zones. Moreover, the total distance moved during the 10 min session was analyzed and the number of fecal boli dropped was counted at the end of each trial.

### Statistical analysis

The aim of the present study was to compare a standardized design with a heterogenized design to examine whether they differ with respect to the reproducibility of strain differences across laboratories, and sample size was determined by the minimum number of animals need for this purpose. For each factor combination of the heterogenized design we used only one cage per strain (the absolute minimum), with 4 mice in each cage (in total n = 16 mice per strain, experimental design, and laboratory). We considered 4 mice per cage the absolute minimum to allow us to compare within-cage variance with between-cage variance as an important control measure to assess whether heterogenization had worked. Moreover, we considered 6 labs sufficient to obtain a reasonable estimate of the effect of heterogenization on reproducibility of behavioral strain differences.

Except for Utrecht, where one animal had died during the test phase, data recordings were complete. However, for 21 out of the 384 animals we had to exclude single values from the final analysis. Reasons for exclusion were (i) mice jumping out of the apparatus (especially in the BT), (ii) mice performing stereotypic circling in the open-field arena, and (iii) problems with video tracking. These missing values were replaced by series means.

All data were analyzed using General Linear Models (GLM). To meet the assumptions of parametric analysis, residuals were graphically examined for homoscedasticity and outliers, and, when necessary, the raw data were transformed using square-root, logarithmic or angular transformations (for a list of transformations see Table 3). For the analysis we selected 29 behavioral measures from the five behavioral tests, including common measures of activity, anxiety and exploratory drive, 20 of which were automatically recorded using specialized software (Table 3).

**Table 3:** Complete list of behavioral measures used for the analysis and the transformations applied to meet the assumptions of parametric analysis (NT = no transformation, log = log_10_(y+1)-transformed, sqrt = square-root-transformed, angular = arcsin(square-root(y))-transformed).

Behavioral test	Behavioral measure	Transformation
BT	latency to climb over the barrier [s]	log
VPT	latency to fall off the pole [s]	log
EZM	total path moved [cm]	sqrt
	path moved in closed sectors [cm]	NT
	time spent in closed segments [s]	angular
	number of open segment entries	sqrt
	total head dips	sqrt
	protected head dips	NT
	bolus count	sqrt
	total stretched postures	sqrt
	rearing frequency	sqrt
OFT	bolus count	NT
	total path moved [cm]	sqrt
	path moved within centre [cm]	sqrt
	path moved within corners [cm]	NT
	corner time [s]	angular
	centre time [s]	angular
	entries centre	NT
	entries corners	NT
NOT	bolus count	NT
	total path moved [cm]	sqrt
	path moved within wall zone [cm]	sqrt
	path moved within exploration zone 1 [cm]	sqrt
	path moved within exploration zone 2 [cm]	sqrt
	exploration frequency 1 (zone Ø 15 cm)	sqrt
	exploration frequency 2 (zone Ø 25 cm)	sqrt
	exploration time 1 (zone Ø 15 cm) [s]	sqrt
	exploration time 2 (zone Ø 25 cm) [s]	sqrt
	wall time [s]	angular

In a first step, we determined mean strain differences ( = mean C57BL/6NCrl mice - mean DBA/2NCrl mice) for all 29 behavioral measures to compare variation among the six laboratories for the standardized and the heterogenized design.

Next, we analyzed the results of each laboratory separately (laboratory-specific analysis) as if each experiment had been conducted independently and assessed the main effect of ‘strain’ on each of the 29 behavioral measures using a GLM split by experimental design. Based on the 2×2 factorial design of the heterogenized condition, and to account for microenvironmental differences due to cage position in the rack, each experiment was divided into the four blocks of cage pairs, and ‘block’ included as a blocking factor in the GLM: y = strain + block. Including ‘block’ as a blocking factor in the GLM allowed us to control for between-block variation, thereby reducing variance in the data and increasing test sensitivity [52], [53].

To explore the difference between the two experimental designs in the variation among laboratories (lab) further, we analyzed the two experimental designs separately using the GLM: y = strain +lab + strain× lab. We then compared the resulting F-ratios of the ‘strain-by-lab’ interaction term between the two experimental designs using a second GLM blocked by behavioral measure: y = experimental design+behavioral measure.

The rationale for using F-ratios for this comparison was twofold, namely (i) that F-ratios are scale invariant, so the different scales of the different test measures became unimportant, and (ii) that F-ratios in a GLM have a discrete null hypothesis (F = 1) which we could test against. The latter is because F-ratios reflect ‘variance components’, so we can think of the true variance of any factor as being = variance due to that factor+residual variance. Thus, in a GLM, if the variance due to the factor under test is 0, then the F-ratio will ideally be 1 (because F-ratio = (variance due to the factor + residual variance)/residual variance). Therefore, if the average F-ratio of the ‘strain-by-laboratory’ interaction term were equal to 1, this would mean that strain differences did not vary between laboratories, which would essentially be the same as perfect reproducibility. To test this statistically, we used a post-hoc t-test of the null hypothesis that F equals 1.

In the GLM used to determine variation among the six laboratories (y = strain + lab + strain× lab), the residual variance accounts for all the within-laboratory variance (except variance due to ‘strain’). However, the residual variance of this model reflects various aspects of within-laboratory variance, including variation due to the heterogenization factors, cage position in the rack, and individual differences. To determine how exactly heterogenization influenced between-experiment variation, we therefore calculated an additional GLM that included ‘block’ and the interaction between ‘strain’ and ‘block’ as factors: y = strain +lab + block(lab) + strain× lab + strain× block(lab). Including ‘block’ and the ‘strain-by-block’ interaction (with ‘block’ nested within ‘lab’) in the GLM, allowed us to calculate an additional F-ratio by dividing the mean squares (MS) of the ‘strain-by-lab’ interaction by the MS of the ‘strain-by-block’ interaction (F = MS(strain×lab)/MS(strain×block(lab))). This F-ratio reflects the partitioning of the strain-by-block variance among all 24 blocks in the six laboratories into variance among blocks of different laboratories (i.e. between-laboratory variation), and variance among blocks within the same laboratory (i.e. within-laboratory variation). It therefore represents an ideal measure to determine how heterogenization affected within-experiment variation relative to between-experiment variation [34]. Our prediction was that this ratio will be smaller for the heterogenized design, and ideally = 1. If it were equal to 1 or lower, this would mean that heterogenization generated as much or even more variance between the four blocks within a laboratory as exists between laboratories. All statistical tests were conducted using the software package SPSS/PASW (version 17.0 for Windows).

## Results

### Effects of strain and laboratory

**Figure 3:**
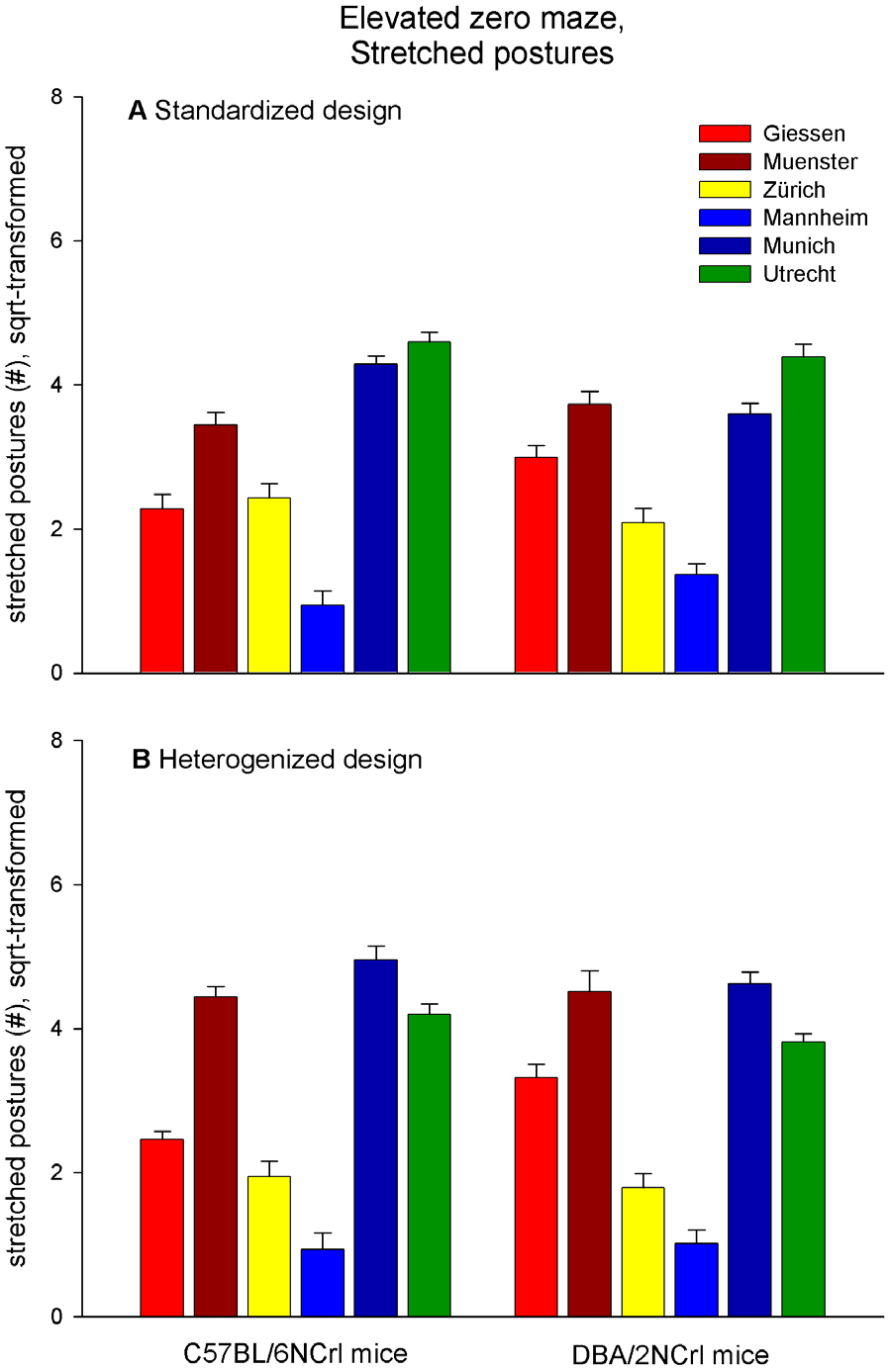
Number of stretched postures on the elevated zero maze shown by C57BL/6NCrl and DBA/2NCrl mice. Data are presented as means (+ s.e.m., square-root-transformed, n = 16/strain and laboratory). The example illustrates large effects of the laboratory in the standardized (**A**) and heterogenized (**B**) design. Moreover, the direction of strain difference differed between Giessen and Munich in the standardized design.

**Figure 4:**
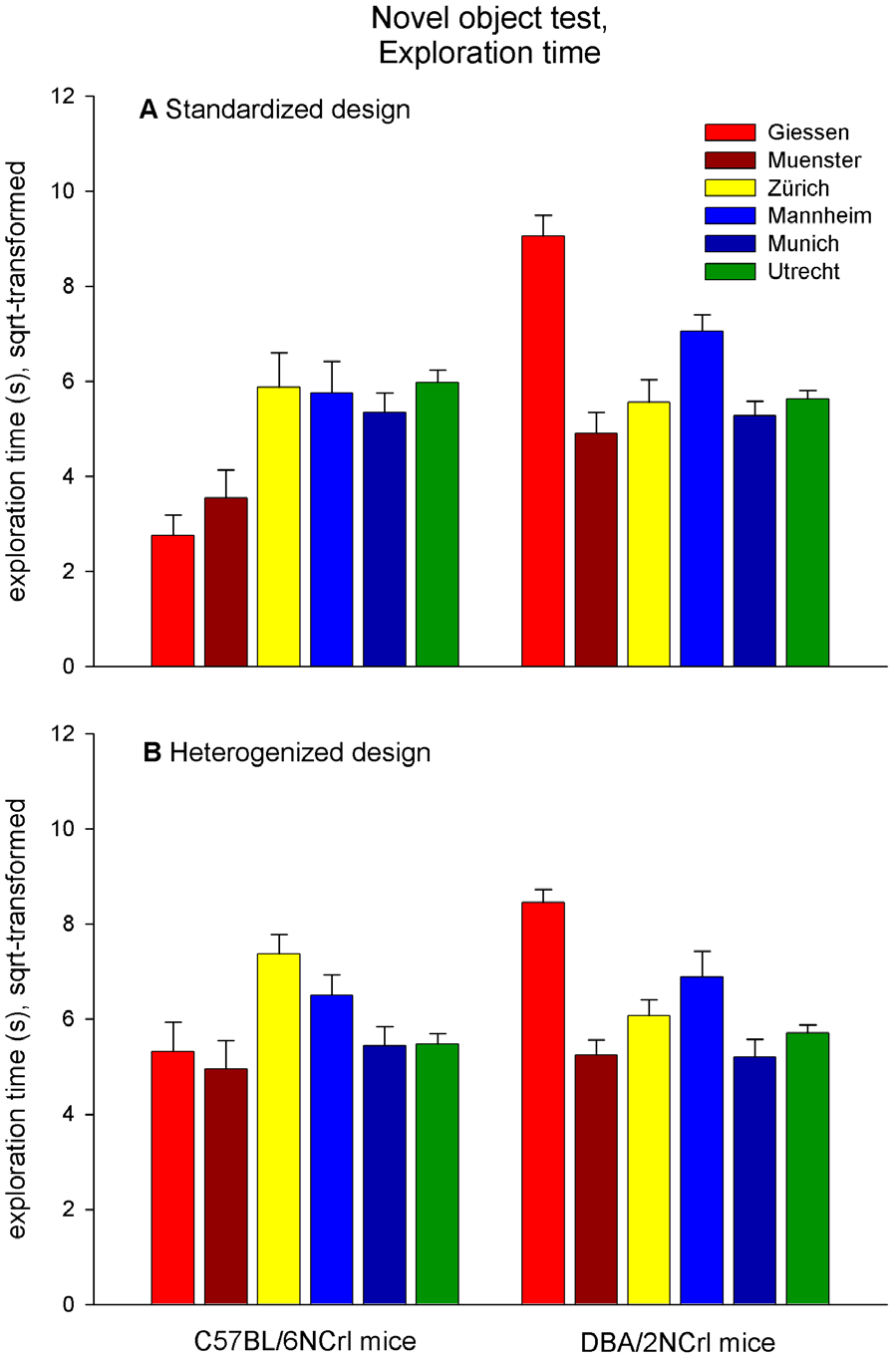
Object exploration time in the novel object test shown by C57BL/6NCrl and DBA/2NCrl mice. Data are presented as means (+ s.e.m., square-root-transformed, n = 16/strain and laboratory). The example illustrates large effects of strain and laboratory in the standardized (**A**) and heterogenized (**B**) design. Moreover, the direction of strain difference differed between Giessen and Zürich in the heterogenized design.

Regardless of the experimental design, significant and, in some cases, large main effects of ‘laboratory’ and ‘strain’ were found for nearly all variables (Table 4). As expected, the absolute values measured were quite variable among laboratories (see Fig. 3 and Fig. 4). Such additive effects of the laboratory, however, occurred in all five behavioral tests, including measures of activity (e.g. total path moved in the open field), exploration (e.g. novel object exploration time and frequency), and anxiety (e.g. time spent in and frequency of entering the centre in the open field; Table 4). For example, mice tested in Muenster were, on average, less active than those tested in other labs (measured by ‘total path moved in the open field test’ or ‘total path moved in the novel object test’). In particular, the number of stretched postures on the elevated zero maze varied most remarkably among the six laboratories (see Fig. 3).

**Table 4:** F-ratios of all behavioral measures for the main effects of ‘strain’ and ‘laboratory’ based on the GLM (split by experimental design): y = strain+laboratory+strain×laboratory; p≤0.001***, p≤0.01**, p≤0.05*, p>0.05 NS.

	Strain				Laboratory				Strain×Laboratory			
	Standardized		Heterogenized		Standardized		Heterogenized		Standardized		Heterogenized	
**latency to climb over barrier [s], BT**	22,575	***	14,080	***	3,988	**	3,022	*	2,526	*	2,803	*
**latency to fall off [s], VPT**	113,205	***	38,735	***	2,704	**	1,918	NS	1,630	NS	,509	NS
**total path moved [cm], EZM**	6,811	**	10,683	***	30,542	***	15,480	***	11,197	***	14,467	***
**path moved in closed sectors [cm], EZM**	0,584	NS	0,931	NS	33,348	***	17,354	***	6,364	***	13,981	***
**time in closed segments [s], EZM**	84,642	***	35,154	***	12,580	***	7,451	***	5,236	***	2,027	NS
**open segment entries, EZM**	56,711	***	31,446	***	11,333	***	0,861	NS	10,182	***	2,867	*
**total head dips, EZM**	106,193	***	61,963	***	2,982	*	6,613	***	3,353	**	3,137	**
**protected head dips, EZM**	42,265	***	38,389	***	1,801	NS	8,978	***	2,166	NS	3,101	*
**bolus count, EZM**	105,702	***	76,452	***	1,600	NS	9,820	***	2,839	*	3,874	**
**total stretched postures, EZM**	0,131	NS	0,050	NS	122,767	***	151,486	***	5,744	***	3,376	**
**number of “rearing”, EZM**	8,715	**	4,870	*	18,002	***	3,692	**	1,469	NS	4,434	***
**bolus count, OFT**	25,920	***	36,667	***	0,920	NS	6,600	***	2,033	NS	4,136	**
**path centre [cm], OFT**	48,366	***	10,774	***	37,967	***	46,303	***	6,160	***	7,372	***
**path corner zone [cm], OFT**	108,431	***	74,080	***	119,952	***	134,583	***	3,028	*	1,977	NS
**total path moved [cm], OFT**	2,255	NS	1,177	NS	39,026	***	41,679	***	5,441	***	4,942	***
**corner time [s], OFT**	88,109	***	13,775	***	34,907	***	43,196	***	3,863	**	7,921	***
**centre time [s], OFT**	92,615	***	49,561	***	36,893	***	43,855	***	3,450	**	3,161	**
**entries centre, OFT**	10,492	***	8,338	**	36,579	***	47,698	***	10,515	***	10,290	***
**entries corner zone, OFT**	24,171	***	19,427	***	30,691	***	26,147	***	11,699	***	4,669	***
**bolus count, NOT**	48,835	***	41,776	***	6,836	***	7,038	***	1,016	NS	2,666	*
**total path moved [cm], NOT**	15,087	***	14,049	***	44,393	***	20,109	***	4,068	**	3,243	**
**path wall zone [cm], NOT**	17,781	***	30,000	***	42,941	***	29,831	***	3,026	*	1,544	NS
**path exploration zone 1 [cm], NOT**	78,443	***	52,096	***	9,794	***	13,029	***	8,301	***	3,960	**
**path exploration zone 2 [cm], NOT**	34,177	***	8,186	**	11,888	***	8,660	***	17,232	***	4,899	***
**exploration frequency 1, NOT**	100,276	***	49,733	***	2,755	*	2,868	*	14,347	***	6,363	***
**exploration frequency 2, NOT**	50,252	***	19,235	***	6,223	***	2,956	*	14,259	***	5,762	***
**exploration time 1 [s], NOT**	88,788	***	75,483	***	10,916	***	11,121	***	3,415	**	1,630	NS
**exploration time 2 [s], NOT**	29,488	***	3,355	NS	5,747	***	8,094	***	16,765	***	6,832	***
**wall time [s], NOT**	0,678	NS	0,064	NS	12,360	***	22,463	***	2,322	*	2,477	*

Furthermore, comprehensive analysis of all data revealed strong differences between C57BL/6NCrl and DBA/2NCrl mice in all five behavioral tests (Table 4). Depending on the specific laboratory, however, the direction of strain differences varied for some behavioral measures. For example, on the elevated zero maze DBA/2NCrl mice showed more stretched postures than C57BL/6NCrl mice in Giessen (standardized design: F_1,27_ = 16.050, p<0.001; heterogenized design: F_1,27_ = 16.077, p<0.001), but fewer in Munich (standardized design: F_1,27_ = 12.949, p<0.001), while they did not differ in Muenster, Zürich, and Utrecht (Fig. 3). In the novel object test, DBA/2NCrl mice explored the novel object much longer than C57BL/6NCrl mice in Giessen (standardized design: F_1,27_ = 101.067, p<0.001; heterogenized design: F_1,27_ = 24.892, p<0.001), while they explored it shorter in Zürich (heterogenized design: F_1,27_ = 5.760, p<0.05) (Fig. 4). For most behavioral measures, however, the laboratory environment was critical in determining the size rather than the direction of strain effects.

### Standardization versus heterogenization

#### Between-experiment variation

**Figure 5:**
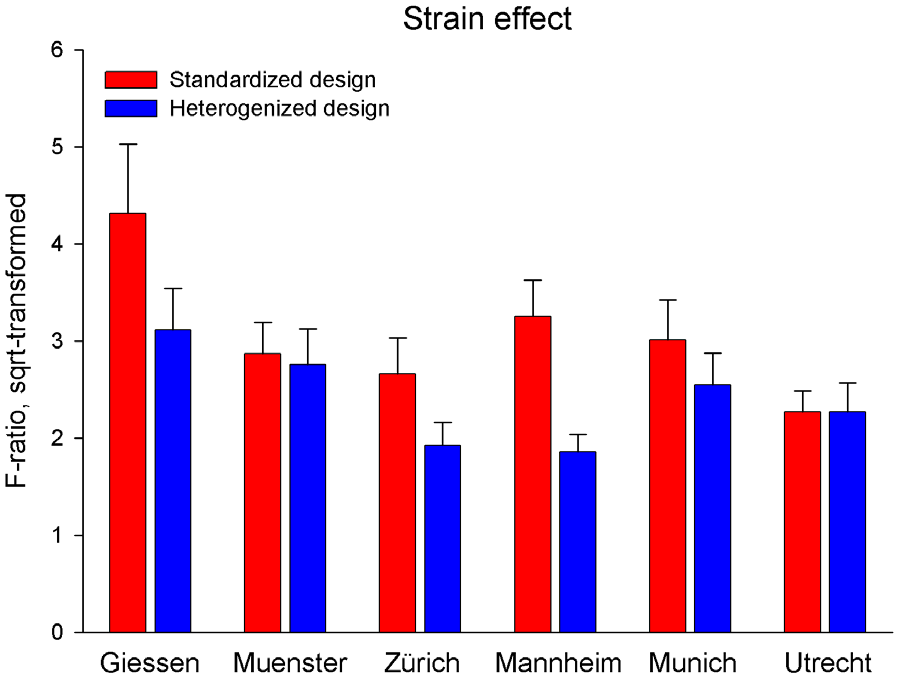
Variation of strain main effects across the six laboratories in both designs. For each laboratory and experimental design, the main effect of ‘strain’ was separately calculated and displayed in terms of the mean F-ratio (+ s.e.m., square-root-transformed) across all 29 behavioral measures. Although the strain effect varied considerably among laboratories in the heterogenized design, the standardized design produced even more variable outcomes. Moreover, average F-ratios for ‘strain’ were considerably higher in the standardized design, indicating that treatment effects may be systematically overestimated by standardization.

To explore the effect of the experimental design on the reproducibility of behavioral strain differences, the effect of ‘strain’ on each of the 29 behavioral measures was assessed separately for each laboratory. Although the average effect of ‘strain’ varied considerably among the six laboratories in the heterogenized design, the standardized design produced even more variable outcomes (Fig. 5). Moreover, the average F-ratios of the ‘strain’ effect were considerably larger in the standardized design (Fig. 5).

**Figure 6:**
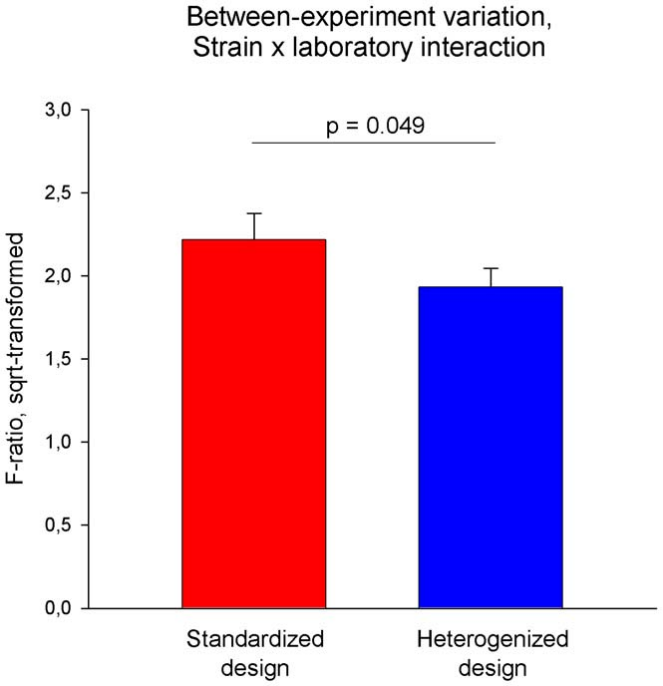
Variation between laboratories in the standardized and in the heterogenized design. The variation in strain differences is displayed as mean F-ratios (+ s.e.m.) of the ‘strain-by-laboratory’ interaction term calculated for 29 behavioral measures. F-ratios were determined separately for the two experimental designs, square-root-transformed to meet the assumptions of parametric analysis, and then compared using a GLM blocked by ‘behavioral measure’. F-ratios of the ‘strain-by-laboratory’ interaction terms were significantly lower in the heterogenized design (F_1,28_ = 4.222, p = 0.049), indicating lower between-experiment variation.

**Figure 7:**
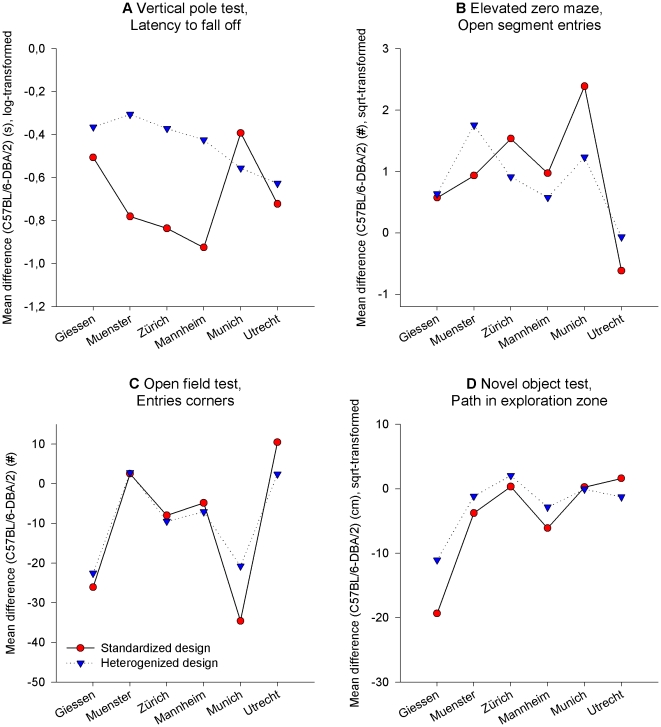
Variation of mean strain differences in the standardized and heterogenized design across the six laboratories. Four examples of selected behavioral measures from four of the five behavioral tests are displayed: (**A**) Latency to fall off the pole in the vertical pole test, (**B**) number of open segment entries on the elevated zero maze, (**C**) number of corner entries in the open field test and (**D**) path travelled within the exploration zone in the novel object test. Strain differences varied considerably between laboratories in both designs, but were somewhat more consistent in the heterogenized design. Each laboratory tested 16 mice per strain for each experimental design.

To confirm these findings statistically, we used the GLM y = strain + lab + strain × lab (see Statistical Analysis) to determine the F-ratios of the ‘strain-by-lab’ interaction term for each of the 29 behavioral measures that were then compared between the two experimental designs. Indeed, these F-ratios were significantly smaller in the heterogenized design (F_1,28_ = 4.222, p = 0.049), indicating improved reproducibility of strain differences among laboratories in the heterogenized design (Fig. 6). However, including ‘block’ in the GLM (y = strain + lab + block(lab) + strain× lab + strain × block(lab)) weakened this effect to a non-significant trend (F_1,28_ = 3,405, p = 0.076), indicating that part of the effect was due to cage position, independent of the heterogenization factors. Moreover, in both designs the average F-ratio was significantly different from 1 (t-test of the null hypothesis that F = 1: standardized design: T_28_ = 7.660, p<0.001; heterogenized design: T_28_ = 8.214, p<0.001), demonstrating that strain effects varied substantially among laboratories in both designs (Fig. 6). Further graphical examination of the mean strain differences across the six laboratories confirmed this, although strain differences were somewhat more consistent in the heterogenized design (Fig. 7).

#### Within-experiment variation

To assess whether improved reproducibility in the heterogenized design was caused by heterogenization shifting variation from between-experiment variation to within-experiment variation, within-experiment variances were averaged across the six laboratories and compared between the two designs for each of the 29 behavioral measures. The average within-experiment variance was larger in 23 out of 29 measures in DBA/2NCrl mice and in 18 out of 29 measures in C57BL/6NCrl mice, suggesting that heterogenization systematically shifted variance from between-experiment to within-experiment variation.

**Figure 8:**
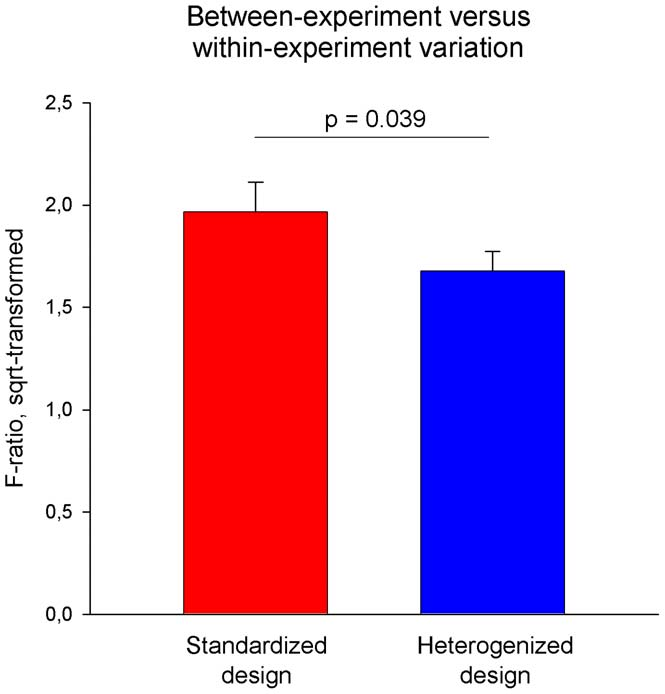
Between-experiment variation versus within-experiment variation. To assess the relative weight of between-laboratory variation versus within-laboratory variation, an F-ratio was calculated that reflects the partitioning of the ‘strain-by-block’ variance between all 24 blocks of one experimental design into variance due to variation between laboratories and variance due to variation within laboratories. For this, the mean squares of the ‘strain-by-laboratory’ interaction term were divided by the mean squares of the ‘strain-by-block’ interaction term. Data are displayed as mean F-ratios (+ s.e.m.; square-root-transformed) across all 29 behavioral measures for both conditions. F-ratios were significantly smaller in the heterogenized design (F_1,28_ = 4.678, p = 0.039), demonstrating that heterogenization increased within-experiment variation relative to between-experiment variation.

To confirm this statistically, we used the GLM y = strain + lab + block(lab) + strain× lab + strain × block(lab) and calculated the F-ratio of the ‘strain-by-lab’ interaction term divided by the ‘strain-by-block’ interaction term (see Statistical Analysis). These F-ratios were significantly smaller in the heterogenized design (F_1,28_ = 4.678, p = 0.039, Fig. 8), demonstrating that heterogenization did indeed increase within-experiment variation (variance among blocks of the same laboratory) relative to between-experiment variation (variance among blocks of different laboratories).

## Discussion

### Strain effects

C57BL/6 and DBA/2 mice, two of the most widely used inbred strains of laboratory mice, are known to differ markedly in many behavioral tasks [54]–[59]. Therefore, it was not surprising to find significant and often large strain differences in almost all behavioral measures assessed in the present study. In line with previous studies, C57BL/6NCrl mice generally showed less anxiety-related behavior than DBA/2NCrl mice [55], [56]. General levels of locomotor activity as measured by the total path moved in the tests, however, did not differ much between the two strains, although C57BL/6 mice are considered to be more active than DBA/2 mice [55], [56].

### The impact of the laboratory environment

We also found considerable differences in the absolute values measured in different laboratories, confirming previous findings [13], [22], [35]. In particular, the frequency of stretched postures on the elevated zero maze differed markedly among laboratories. Such large differences in the absolute values may be typical for manually recorded measures and reflect experimenter-dependent variability, highlighting the importance of inter-observer reliability training [60], [61] and the value of automated data recording [62], [63]. However, such additive differences among laboratories do not normally threaten the validity of strain differences. For example, Munich and Muenster used ANYmaze (Stoelting Co.) for video-tracking of open field and elevated zero maze performance, while the other four laboratories used two different versions of EthoVision (Noldus Information Tecnology). Differences in software functioning may indeed explain some variation in the absolute values measured, but should not affect the size and direction of strain differences.

Despite the marked phenotypic differences between these two strains, however, we also found variation in the direction of strain differences among laboratories in some measures, indicating that the same test conducted in different laboratories may lead to fundamentally different conclusions. Such dramatic strain-by-laboratory interactions may arise when different strains respond differently to the specific environmental or testing conditions of the different laboratories. When using strains that are phenotypically less distinct as is often the case when transgenic strains are compared with wild-type strains [64], this may actually be the norm rather than an exception given that many phenotypic states are highly dependent on environmental conditions [11], [19], [30], [57], [65]. In the present study, some aspects of the housing and testing conditions were equated between laboratories (e.g. supplier, testing order, position of cages within the rack), while others remained laboratory-specific (e.g. local room architecture, tracking software, experimenter, time of testing, handling and identification method). However, because both additive and non-additive laboratory effects may arise from any or all of these laboratory-specific aspects, any further explanation of these effects in terms of single factors is impossible.

### Reproducibility of the results

Poor reproducibility is typically caused by interactions of genotype with the specific laboratory conditions. To avoid this, scientists are generally advised to strengthen efforts of standardization both within and between laboratories [23], [27], [66], [67]. However, attempts to avoid poor reproducibility by more rigorous standardization are misleading. If fully effective, standardization within laboratories would decrease variation within study populations to zero [28], and therefore, each experiment would turn into a single-case study with zero information gain, producing statistically significant, but irrelevant results that lack generality under even slightly different conditions [28], [29]. Indeed, the average F-ratios of the ‘strain’ effect were considerably larger in the standardized design, indicating that standardization may systematically overestimate strain main effects. The obvious reason for this is that interactions between strain and the laboratory-specific conditions are mistaken for strain main effects [32], [34].

Instead of rigorous standardization, we proposed systematic variation of experimental conditions to render populations of experimental animals more heterogeneous, thereby improving the external validity of results across the unavoidable variation among laboratories [32], [34]. The findings reported here are somewhat ambiguous with respect to the efficacy of heterogenization in improving reproducibility. Thus, although heterogenization did have an effect in the predicted direction, this effect was rather weak, and both heterogenization and standardization resulted in relatively poor reproducibility. The reason for this might be that either heterogenization did not work with our selection of behavioral measures or that the type of heterogenization employed here was not effective enough.

Both the reproducibility of behavioral measures and the effect of heterogenization on their reproducibility may vary depending on the exact selection of measures. However, heterogenization should have the weakest effect on those measures that are least sensitive to environmental conditions. Such measures should also be highly reproducible under both standardized and heterogenized conditions. The present analysis was based on a selection of 29 behavioral measures from five tests that are widely used in behavioral phenotyping or drug screening studies. The fact that nearly all of these measures varied considerably among laboratories suggests that they were highly sensitive to environmental conditions. Therefore, our selection of measures is unlikely to account for the relatively weak improvement of reproducibility by heterogenization. Instead, our findings suggest that the study populations generated within laboratories by the form of heterogenization employed here did not adequately represent the range of variation between the six laboratories. The reason for this may be that age and cage enrichment were poor heterogenization factors, or that the specific levels of these factors were not different enough to induce sufficient variation in behavioral phenotypes.

### Efficacy of heterogenization

Our choice of heterogenization factors was based on practical considerations and on studies demonstrating that both age and enrichment affect, and interact with, a variety of potential outcome measures [36]–[42], [68], [69]. It is possible that more distinct levels of these factors would have produced stronger effects. Moreover, the pretest housing period was limited to three weeks for logistic reasons. Perhaps a longer exposure of the mice to the laboratory-specific conditions would have strengthened the effects of the heterogenization factors. On the other hand, more extreme variation of age and enrichment, or a longer housing period would have rendered heterogenization less practicable. This raises the question whether other factors might be more effective in heterogenization.

Many of the measures obtained from behavioral tests are highly sensitive to test conditions. Paylor [33] suggested running experiments in several batches tested on different days. While this may be an effective strategy since test conditions are likely to vary from day to day, it is not a well controlled strategy, and efficacy may vary greatly both within and between laboratories. Alternatively, specific factors of the test conditions may be used for systematic heterogenization similar to age and enrichment in the present study. For example, test time, background noise, and illumination level have all been shown to affect test responses [70]–[73]. It is possible that heterogenization through factors of the test conditions would be more effective because their effects on the animals' test responses are more immediate.

Taken together, the fact that the results varied greatly between laboratories in both designs confirms the need for effective heterogenization strategies to guarantee reproducible test results. Therefore, further research is needed to identify and validate factors that exert sufficiently strong effects on behavioral phenotypes. Because poor reproducibility occurs throughout animal experimentation, this research should aim at heterogenization strategies that are either applicable to a wide range of different studies or are specifically tailored to specific types of studies.

### Conclusions

Despite strong effects of the laboratory on nearly all behavioral measures in both designs, the findings of this study confirm our earlier findings [32], [34], and indicate that systematic heterogenization may also improve reproducibility in a real multi-laboratory situation. By systematically increasing within-experiment variation relative to between-experiment variation, heterogenization tended to improve reproducibility compared to standardization. However, the ratio of between-experiment to within-experiment variation was far greater than 1 in both designs, indicating that between-laboratory variation was substantially greater than within-laboratory variation. This underscores the need for more powerful heterogenization strategies to guarantee reproducibility of results across the large variation among different laboratories.
